# Retrospective analysis of the sex chromosomal copy number variations in 186 fetuses using single nucleotide polymorphism arrays

**DOI:** 10.3389/fgene.2022.997757

**Published:** 2022-12-01

**Authors:** Haiwei Wang, Bin Liang, Yan Wang, Hailong Huang, Na Lin, Liangpu Xu

**Affiliations:** Fujian Key Laboratory for Prenatal Diagnosis and Birth Defect, Medical Genetic Diagnosis and Therapy Center, Fujian Maternity and Child Health Hospital, Fujian Medical University, Fuzhou, Fujian, China

**Keywords:** sex chromosomal abnormalities, copy number variations, prenatal diagnosis, single nucleotide polymorphism array, Turner syndrome

## Abstract

Sex chromosomal abnormalities are associated with multiple defects. However, the types of sex chromosomal abnormalities during pregnancy in Fujian Province, China, are not recorded. In this retrospective analysis, we showed the sex chromosomal abnormalities of 186 fetuses, including 162 cases of X chromosomal abnormalities and 22 cases of Y chromosomal abnormalities in Fujian Province. We detected 73 cases of Turner syndrome, 24 cases of triple X syndrome, 37 cases of Klinefelter syndrome, and 14 cases of XYY syndrome. It was observed that 67.3% fetuses with classic Turner syndrome had their growth arrested. Moreover, we found 21 cases of mosaic Turner syndrome, 3 cases of mosaic Triple X syndrome, 2 cases of mosaic Klinefelter syndrome, and 1 case of mosaic XYY syndrome. Furthermore, 37 cases of large scales of sex chromosomal deletions/duplications were detected, including 30 cases of X chromosomal deletions/duplications and 7 cases of Y chromosomal deletions/duplications. Parent-of-origins of five cases of sex chromosomal deletions/duplications were determined. One case was with *de novo* X chromosomal variations, while the sex chromosomal deletions/duplications in other four cases were inherited from their parents. Overall, our results presented a detailed manifestation of sex chromosomal abnormalities of 186 fetuses in Fujian Province and suggested the important roles of single nucleotide polymorphism (SNP) array analysis in the prenatal diagnosis of sex chromosomal abnormalities. Also, determining the parent-of-origins of the deletions/duplications was critical for the prenatal diagnosis of sex chromosomal abnormalities.

## Introduction

The integrity of sex chromosomes, including X and Y chromosomes, is critical for normal embryonic development. Sex chromosomal abnormalities are associated with multiple defects, including sexual organ dysplasia, low reproductive ability, and infertility (Leggett et al., 2010). Turner syndrome (45, X), triple X syndrome (47, XXX), Klinefelter syndrome (47, XXY), and XYY syndrome (47, XYY) are common aneuploidies of sex chromosomes (Nielsen and Wohlert, 1991). Moreover, large scales of sex chromosomal deletions/duplications are also detected in abnormal fetuses and associated with different phenotypes (Zhang et al., 2018). With the increase in the maternal age at conception, the incidence of sex chromosomal abnormalities during pregnancy is growing every year (Lei and Dong, 2019; Li et al., 2021). So, for pregnant women, prenatal diagnosis about sex chromosomal abnormalities is deeply needed.

Karyotype analysis and non-invasive prenatal testing or screening (NIPT or NIPS) (Deng et al., 2019; Deng et al., 2021; Shi et al., 2021) could be used to detect or screen the aneuploidies of sex chromosomes. However, in mosaic cases, sex chromosomal abnormal cells and normal cells exist together. Classic karyotype analysis and NIPT analysis are limited in determining mosaic sex chromosomal abnormalities (Ma et al., 2021). On the contrary, the single nucleotide polymorphism (SNP) array is validated in detecting chromosomal syndromes, mosaic chromosomal syndromes, and chromosomal deletions/duplications, with high accuracy and high resolution (Samango-Sprouse et al., 2013).

From 2019 to 2022, more than 10,000 cases of SNP arrays were conducted in Fujian Maternity and Child Health Hospital. In this study, we retrospectively analyzed the SNP arrays of 186 early fetuses with sex chromosomal copy number variations (CNVs). Among them, 72 cases of Turner syndrome, 24 cases of triple X syndrome, 37 cases of Klinefelter syndrome, and 15 cases of XYY syndrome were detected. Moreover, mosaic sex chromosomal CNVs were determined. Sex chromosomal deletions/duplications were found in 37 cases and parent-of-origins of the deletion/duplications were determined in five cases. Overall, our results presented a detailed manifestation of sex chromosomal abnormalities during pregnancy in Fujian Province and sex chromosomal abnormalities using parental samples.

## Materials and methods

### Clinical patients

This was a retrospective analysis of 186 fetuses with sex chromosomal abnormalities at the Medical Genetic Diagnosis and Therapy Center, Fujian Maternity and Child Health Hospital, including 74 male fetuses and 112 female fetuses. The mean maternal age at conception was 30.98 years old. The clinical conditions of the fetuses were examined by level 2/3D ultrasound. Genetic counseling and prenatal diagnosis were provided for all pregnant women. Informed consent for the SNP analysis from each patient was obtained. All analyses were approved by the Fujian Maternal and Child Health Hospital ethics committee (ID No. 2020KY113).

### Chromosomal microarray analysis

In total, 119 amniotic fluid samples, 53 chorionic villi, 13 cord blood samples, and one skin tissue were collected by the nurse in accordance with the Declaration of Helsinki. Fetal DNA was extracted using a QIAGEN DNA Mini Kit, according to the manufacturer’s instructions. SNP array experiments were carried out according to Affymetrix CytoScan 750K array standard protocols. The Affymetrix CytoScan 750K array includes 550,000 CNV probes and 200,000 SNP probes for the CNV analysis and is wildly used for prenatal diagnosis. The microarray was scanned using a GeneChip Scanner 3000 system and annotated using Chromosome Analysis Suite (ChAS) software based on the hg19 human reference sequence. The chromosomal abnormalities in each sample were assessed by the American College of Medical Genetics and Genomics (ACMG) guidelines by ChAS software.

### Prenatal diagnosis

Based on the Database of Genomic Variants (DGV), Online Mendelian Inheritance in Man (OMIM), and Database of Chromosomal Imbalance and Phenotype in Humans Using Ensembl Resources (DECIPHER), the CNVs of sex chromosomes are classified into likely benign and benign, pathogenic variants, likely pathogenic variants, and variants of uncertain clinical significance (VOUS). Peripheral blood from the parents of the fetuses with sex chromosomal deletions/duplications was used to determine the parent-of-origins of the deletions/duplications. The types of CNVs were further determined by the results of the pedigree analysis and SNP arrays.

## Results

### General sex chromosomal abnormalities in our study

**FIGURE 1:**
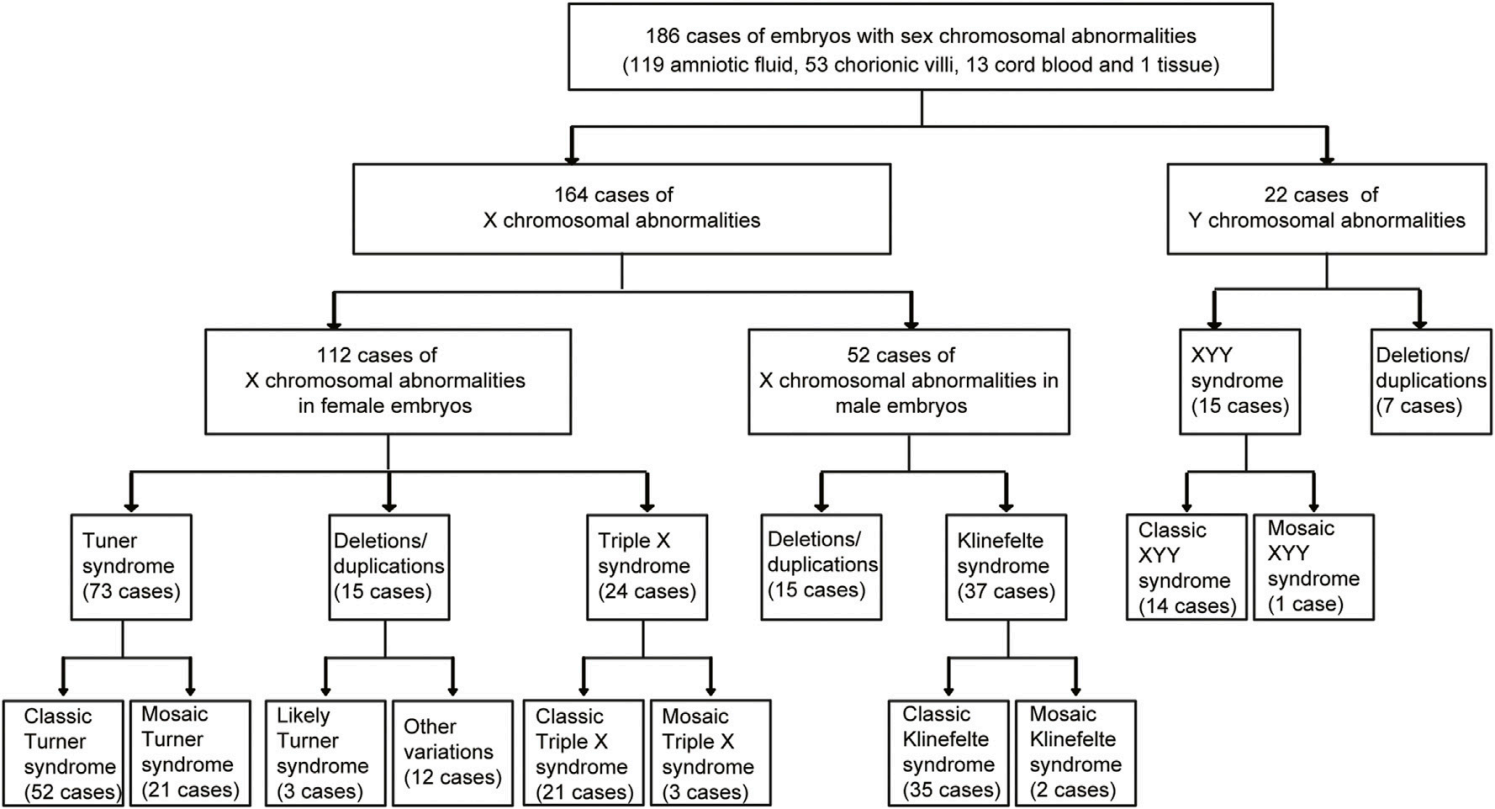
Retrospective analysis of 186 fetuses with sex chromosomal abnormalities.

From 2019 to 2022, 186 fetuses were detected with sex chromosomal abnormalities at Fujian Maternity and Child Health Hospital. The sex chromosomal abnormalities of those 186 samples are shown in Figure 1, including 164 cases of X chromosomal abnormalities and 22 cases of Y chromosomal abnormalities. A total of 112 cases of X chromosomal abnormalities were detected in female fetuses, while 52 cases of X chromosomal abnormalities were detected in male fetuses. Turner syndrome (45, X) and triple X syndrome (47, XXX) are common abnormal manifestations of the X chromosome in female fetuses. We detected 73 cases of Turner syndrome and 24 cases of triple X syndrome (Figure 1). Klinefelter syndrome (47, XXY) was detected in 37 cases of female fetuses. XYY syndrome (47, XYY) is a common abnormal manifestation of the Y chromosome in male fetuses, and 15 cases of XYY syndrome were detected. Moreover, 37 cases of large scales of sex chromosomal deletions/duplications were detected, including 15 cases of X chromosomal deletions/duplications in female fetuses, 15 cases of X chromosomal deletions/duplications in male fetuses, and 7 cases of Y chromosomal deletions/duplications in male fetuses.

In mosaic cases, sex chromosome abnormal cells and normal cells exist together. We found 21 cases of mosaic Turner syndrome out of 73 cases of Turner syndrome. Moreover, three cases of mosaic triple X syndrome, two cases of mosaic Klinefelter syndrome, and one case of mosaic XYY syndrome were detected (Figure 1).

### Clinical phenotypes of fetuses with sex chromosomal abnormalities

Turner syndrome, triple X syndrome, Klinefelter syndrome, and XYY syndrome have diverse phenotypes. The clinical phenotypes of fetuses with classic sex chromosomal aneuploidies are shown in Table 1. We found that 35 (67.3%) fetuses with classic Turner syndrome had their growth arrested. Two cases of classic Klinefelter syndrome also had their growth arrested. However, other sex chromosomal aneuploidies did not affect the early growth of the embryos. Moreover, five cases of classic Turner syndrome were with nuchal translucency (NT) thickening, three cases of classic Turner syndrome were with congenital heart disease, and three cases of classic Turner syndrome were with lymphocystoma. Also, 11 (52.4%) cases of classic triple X syndrome, 25 (71.4%) cases of classic Klinefelter syndrome, and 10 (71.4%) cases of classic XYY syndrome were previously detected with a high NIPT risk of sex chromosomal abnormalities.

**TABLE 1:** Clinical phenotypes of fetuses with classic sex chromosomal aneuploidies.

Ultrasound	Classic Turner syndrome (N = 52)	Classic triple X syndrome (N = 21)	Classic Klinefelter syndrome (N = 35)	Classic XYY syndrome (N = 14)
Embryonic growth arrest	35	0	2	0
NT thickening	5	0	3	1
Congenital heart disease	3	1	1	1
Lymphocystoma	5	0	0	0
Fetal growth restriction	0	1	1	0
Choroid plexus cysts		1	1	0

In contrast with triple X syndrome, Klinefelter syndrome, or XYY syndrome, Turner syndrome had the most mosaic cases. The mosaic CNV values, mosaic percentage, and clinical phenotypes of early fetuses with mosaic Turner syndrome are shown in Table 2. The mosaic CNV values of Turner syndrome ranged from 1.3 to 1.9 and the percentage of the 45, X cell ranged from 10% to 80%. Some embryonic phenotypes of mosaic Turner syndrome were similar to those of classic Turner syndrome, like embryonic growth arrest, NT thickening, and a high NIPT Turner risk (Table 2).

**TABLE 2:** CNVs and percentage of mosaic Turner syndrome.

Case	CNV value	45, X cell (%)	Ultrasound/NIPT
1	Undetermined	10	Embryonic growth arrest
2	Undetermined	30	Embryonic growth arrest
3	1.3	70	Embryonic growth arrest
4	1.8	20	Embryonic growth arrest
5	1.2	80	NT thickening
6	1.3	70	Embryonic growth arrest
7	1.3	70	Embryonic growth arrest
8	1.9	30	Non-structural abnormalities
9	1.7	30	NIPT high Turner risk
10	1.7	30	Fetal echogenic bowel
11	1.5	50	NIPT high Turner risk
12	1.7	30	NIPT high Turner risk
13	1.8	20	Hydronephrosis
14	1.8	20	NIPT high Turner risk
15	1.4	60	NIPT high Turner risk
16	1.86	14	NIPT high Turner risk
17	1.82	18	NIPT high Turner risk
18	1.7	30	Non-structural abnormalities
19	1.77	23	NIPT high Turner risk
20	1.68	32	NIPT high Turner risk
21	1.5	50	Non-structural abnormalities

### X chromosomal deletions/duplications and prenatal diagnosis

Except sex chromosomal aneuploidies, 30 cases of large scales of X chromosomal deletions/duplications were detected in our study, including 15 cases of X chromosomal deletions/duplications in female fetuses and 15 cases of X chromosomal deletions/duplications in male fetuses. Detailed information on the 15 cases of X chromosomal deletions/duplications in female fetuses is shown in Table 3. The X chromosomal deletions/duplications mainly occurred in Xp22.31, Xp22.33, and Xq28 regions. In the female fetuses, five (33.3%) cases were with Xp22.31 deletions, four (26.7%) cases were with Xp22.33 deletions, and two (13.3%) were with Xq28 deletions (Table 3).

**TABLE 3:** X chromosomal deletions/duplications in female fetuses.

Case	Regions (starts–ends)	CNV	Size (Mb)	Number and representative OMIM gene	Prenatal diagnosis
1	Xp22.2 (9,505,232–10,476,941)	3	0.972	5 (*MID1*)	Probably benign
2	Xp22.31 (6,449,836–8,143,509)	1	1.6	8 (*STS*)	VOUS
3	Xp22.31 (6,449,558–8,141,076)	1	1.69	6 (*STS*)	VOUS
4	Xp22.31 (6,449,558–8,141,076)	1	1.69	6 (*STS*)	VOUS
5	Xp22.31 (6,835,778–7834,078)	0	0.998	3 (*STS*)	VOUS
6	Xp22.31 (6,449,558–8,141,076)	1	1.69	5 (*STS*)	VOUS
7	Xp22.33 (168,551–2,958,480)	1	2.79	29 (*SHOX* and *ARSE*)	Pathogenic
8	Xp22.33p11.1 (168,551–58,527,155)	13	58.4	300 (*SHOX* and *ARSE*)	Pathogenic
	Xp11.1q28 (61,882,314–155,233,098)		93.4	411 (*MECP2* and *PLP1*)	
9	Xp22.33p21.3 (168,551–26,023,162)	1.87	25.8	118 (*SHOX* and *STS*)	Pathogenic (likely mosaic Turner)
	Xp21.3q28 (26,031,561–155,233,098)	1.40	129.2	628 (*ARX* and *DMD*)	
10	Xp22.33p22.31 (168,551–8,881,475)	1	8.7	40 (*SHOX*)	Pathogenic (likely mosaic Turner)
	Xp22.31q28 (8,931,445–155,233,098)	1.4	146.3	671 (*MID1* and *HCCS*)	
11	Xp22.33p22.32 (168,551–4,422,774)	1	4.2	31 (*SHOX*)	Pathogenic
12	Xq12 (67,210,899–67,626,475)	3	0.416	1 (*OPHN1*)	VOUS
13	Xq24q25 (118,395,148–125,416,121)	1	7	38 (*UBE2A*, *LAMP2*, and *UPF3B*)	VOUS
14	Xq28 (154,120,632–154,564,050)	1	0.443	6 (*F8*)	VOUS
15	Xq28 (147,550,751–155,233,098)	1	7.68	107 (*F8*)	Pathogenic

The X chromosomal deletions/duplications in the Xp22.31 region were mostly associated with the STS OMIN gene. Loss of the STS gene was associated with multiple defects, including X-linked ichthyosis. X chromosomal deletions/duplications in the Xp22.33 region were mostly associated with the SHOX OMIN gene. SHOX gene deletion can lead to short stature or Leri–Weill dyschondrosteosis disorder. Deletions/duplications in Xp22.31 and Xp22.33 regions were considered pathogenic CNVs in female fetuses (Table 3).

Detailed information on 15 cases of X chromosomal deletions/duplications in male fetuses is shown in Table 4. Similar to female fetuses, the X chromosomal deletions/duplications in male fetuses also occurred in the Xp22.31 and Xp22.33 regions. We found that nine (60%) cases of male fetuses were with Xp22.31 deletions, three (20%) cases were with Xp22.33 deletions, and one was with Xp22.33 duplication (Table 4). Deletions/duplications in the Xp22.31 and Xp22.33 regions were also associated with STS and SHOX OMIN genes and were considered as pathogenic CNVs in male fetuses (Table 4).

**TABLE 4:** X chromosomal deletions/duplications in male fetuses.

Case	Region (starts–ends)	CNV	Size (Mb)	Number and representative OMIM gene	Prenatal diagnosis
1	Xp21.1 (31,987,021–32,181,659)	0	0.195	1 (*DMD*)	VOUS
2	Xp22.12 (20,220,457–20718,134)	2	0.498	1 (*RPS6KA3*)	VOUS
3	Xp22.31 (6,455,361–8,135,568)	0	1.6	5 (*STS*)	Pathogenic
4	Xp22.31 (6,449,836–8,141,076)	0	1.69	6 (*STS*)	Pathogenic
5	Xp22.31 (6,449,836–8,141,076)	0	1.69	6 (*STS*)	Pathogenic
6	Xp22.31 (6,715,163–7,918,931)	0	1.2	4 (*STS*)	Pathogenic
7	Xp22.31 (6,449,836–8,141,076)	0	1.69	5 (*STS*)	Pathogenic
8	Xp22.31 (6,683,449–7,814,664)	0	1.13	3 (*STS*)	Pathogenic
9	Xp22.31 (6,455,151–8135,568)	0	1.68	4 (*STS*)	Pathogenic
10	Xp22.31 (6,455,151–8135,568)	0	1.68	4 (*STS*)	Pathogenic
11	Xp22.31 (6,455,151–8135,568)	0	1.68	4 (*STS*)	Pathogenic
12	Xp22.33 (168,552–1234,634)	0	1.1	5 (*SHOX*)	Pathogenic
13	Xp22.33 (168,551–629,999)	0	0.461	5 (*SHOX*)	Pathogenic
14	Xp22.33 (387,396–629,998	0	0.243	1 (*SHOX*)	Pathogenic
15	Xp22.33p22.32 (2,372,667–5,718,525)	2	3.3	11 (*ARSE*)	Probably benign

### Parent-of-origins of the X chromosomal deletions/duplications

**FIGURE 2:**
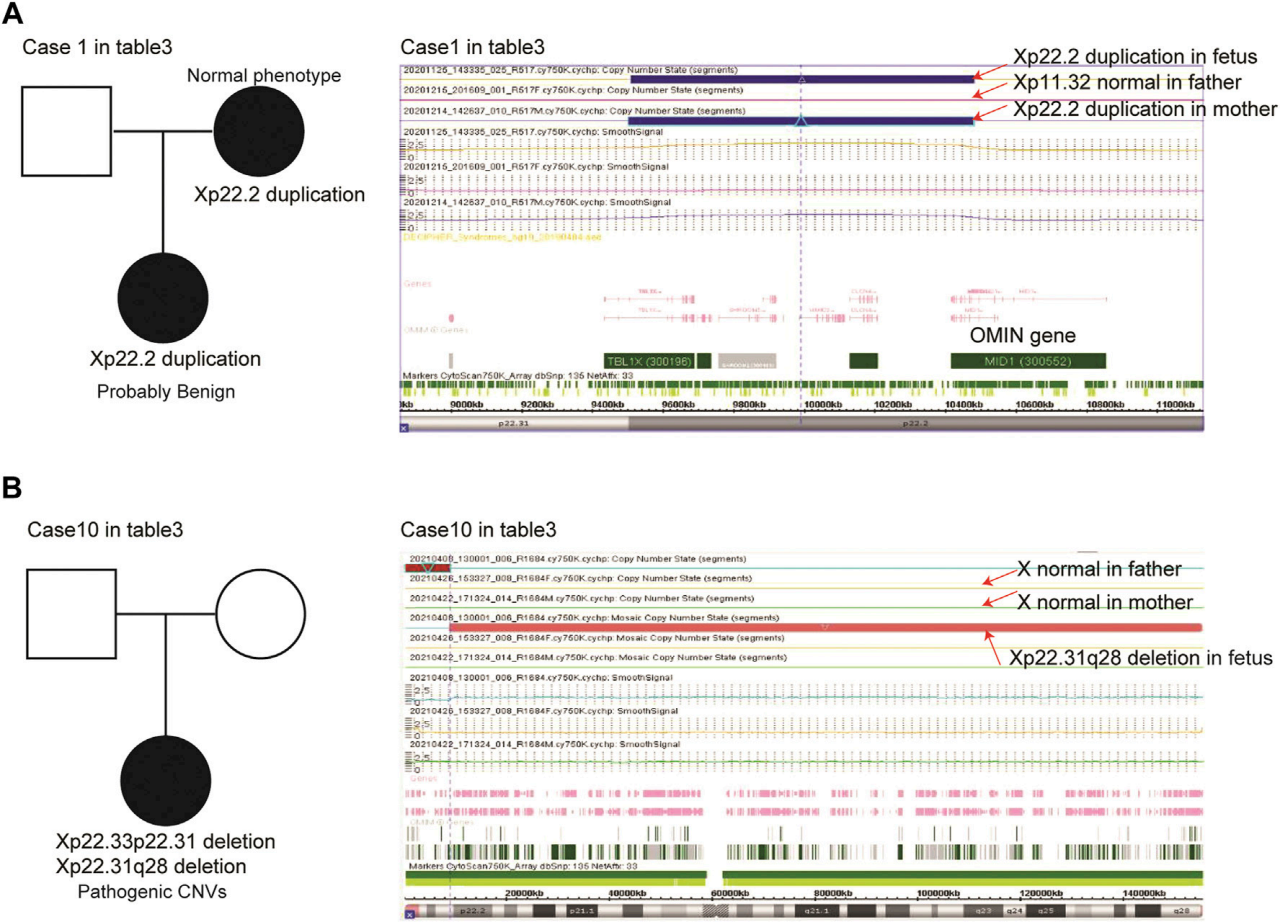
Parent-of-origins of the X chromosomal deletions/duplications in case 1 **(A)** and case 10 **(B)** female fetuses.

Origins of the X chromosomal deletions/duplications in female or male fetuses were determined using parental samples. Xp22.2 duplications were found in case 1 female fetuses (Table 3). After chromosomal analysis of the parents of case 1, we found that her mother but not her father shared similar Xp22.2 duplications and suggested that the Xp22.2 duplications in case 1 female fetuses were inherited from her mother (Figure 2A). Moreover, her mother had normal phenotypes and the Xp22.2 duplications in case 1 were defined as probably benign CNVs.

Xp22.33p22.31 and Xp22.31q28 deletions were found in case 10 female fetuses (Table 3). We found that her parents had no Xp22.33p22.31 or Xp22.31q28 deletions, suggesting that the X chromosomal variations in case 10 were *de novo* alterations (Figure 2B). Larger scales of Xp22.31q28 deletions influenced 671 OMIN genes, including *MID1* and *HCCS* genes, and this alteration was defined as pathogenic CNVs.

**FIGURE 3:**
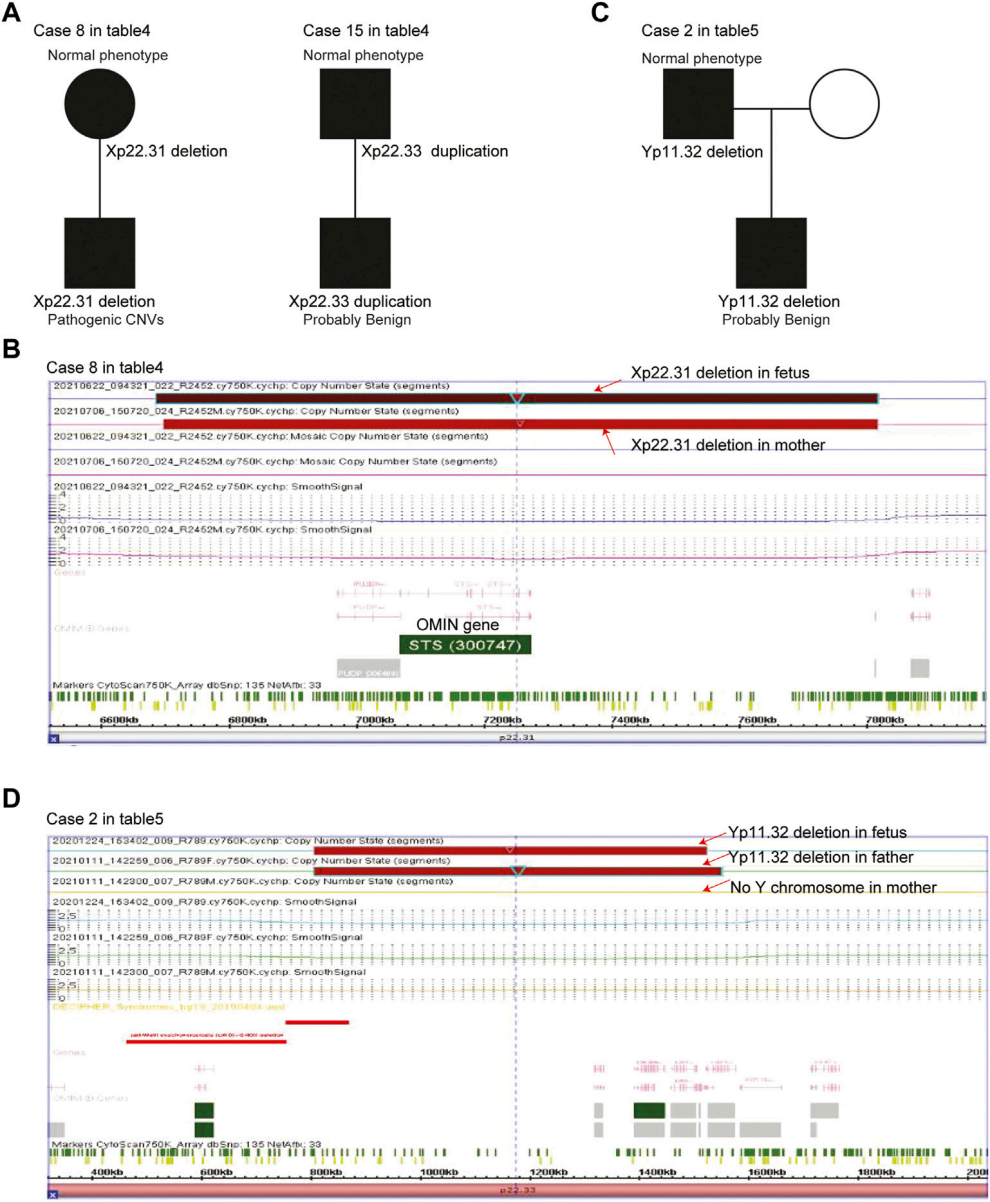
Parent-of-origins of the sex chromosomal deletions/duplications in male fetuses. **(A)** Pedigree of case 8 and case 15 female fetuses with X chromosomal deletions/duplications. **(B)** Xp22.31 deletions in the case 8 male fetus and his mother. **(C)** Pedigree of the case 1 female fetus with X chromosomal deletions/duplications. **(D)** Yp11.32 deletions in the case 1 male fetus and his father.

Parent-of-origins of the X chromosomal deletions/duplications were also determined in case 8 and case 15 male fetuses (Table 4). The Xp22.31 deletions in case 8 male fetuses were inherited from his mother (Figure 3A). The case 8 male fetus and his mother shared similar X chromosomal deletions (Figure 3B). Although his mother had normal phenotypes, the Xp22.31 deletions were known pathogenic CNVs. The Xp22.33 duplications in the case 15 male fetus were inherited from his father. His father had normal phenotypes, and the Xp22.33 duplications in case 15 were defined as probably benign CNVs (Figure 3A).

### Y chromosomal deletions/duplication and prenatal diagnosis

We also detected seven cases of large scales of Y chromosomal deletions/duplications. Detailed information on Y chromosomal deletions/duplications is shown in Table 5, including two cases of Yp11 duplications and five cases of Yq11 deletions. Yq11 included AZFa, AZFb, and AZFc regions. Alterations of these regions were associated with male infertility, and Yq11 deletions/duplications were considered pathogenic CNVs. In the DGV, loss of Yq11.223q11.23 is defined as VOUS.

**TABLE 5:** Y chromosomal deletions/duplications in male fetuses.

Case	Region (starts-ends)	CNV	Size (Mb)	Number and representative OMIM gene	Prenatal diagnosis
1	Yp11.31q11.221 (485,572–18,016,216)	2	17.4	36 (*SRY* and *AZFa*)	Pathogenic
2	Yp11.32 (803,294–1,519,822	0	0.717	8 (*CSF2RA*)	Probably benign
3	Yq11.1q11.23 (13,134,517–28,799,654)	0	15.6	29 (*AZFa*, *AZFb*, and *AZFc*)	Pathogenic
4	Yq11.221 (16,189,079–28,799,654)	2	16	32 (*AZFb* and *AZFc*)	Pathogenic
5	Yq11.222q11.2 (20,094,029–28,398,950)	0	8.3	21 (*AZFb* and *AZFc*)	Pathogenic
6	Yq11.223q11.23 (24,741,034–2,8,372,003)	0	3.6	10 (*AZFc*)	VOUS
7	Yq11.223q11.23 (25,863,808–27,609,692)	0	1.75	6 (*AZFc*)	VOUS

Parent-of-origins of the Y chromosomal deletions/duplications were also determined in case 2. The Yp11.32 deletions in the case 2 male fetus were inherited from his father (Figure 3C). The case 2 male fetus and his father shared similar Y chromosomal deletions (Figure 3D). His father had normal phenotypes, and Yp11.32 deletions in case 2 were defined as probably benign CNVs.

## Discussion

Turner syndrome, triple X syndrome, Klinefelter syndrome, and XYY syndrome are the most common abnormal manifestations of sex chromosomes (Nielsen and Wohlert, 1991). In our study, 122 (65.6%) cases out of 186 cases with sex chromosomal abnormalities were classified into those four subtypes. One missing X chromosome in females (Turner syndrome) was associated with severe defects and with the absence of further fetal development in some cases. Also, 35 (67.3%) fetuses with Turner syndrome had their growth arrested. However, most girls with triple X syndrome grow up healthy and have normal sexual development. An extra X chromosome (Klinefelter syndrome) or Y chromosome (XYY syndrome) in male fetuses usually has no severe defects. Those and our results highlighted the different phenotypes of males or females with sex chromosomal abnormalities (Migeon, 2020).

In our study, more than 40% cases were associated with Turner syndrome. Turner syndrome was correlated with the deletion of one entire X chromosome in all embryonic cells (classic Turner syndrome) or in partial of embryonic cells (mosaic Turner syndrome). We identified 52 cases of classic and 21 cases of mosaic Turner syndrome. The mosaic percentage of Turner syndrome was from 10% to 80%. However, the SNP array could not detect the mosaicism as low as 5% (Zheng et al., 2019) and should be detected by whole-exome sequencing or other technologies (Murdock et al., 2017). Moreover, other X chromosomal abnormalities, like isochromosome Xq, isodicentric Xp, ring X chromosome, or large scales of X chromosomal deletions were also associated with Turner syndrome (Gravholt et al., 2017). Three cases with large scales of X chromosomal deletions were found in our study. However, isochromosome Xq, isodicentric Xp, and ring X chromosome were not determined. Those results highlighted the complexity of Turner syndrome and should be further studied.

In our study, 37 cases of large scales of X chromosomal deletions/duplications were detected, including 30 cases of X chromosomal deletions/duplications and 7 cases of Y chromosomal deletions/duplications. The prenatal diagnosis of those variations was difficult. Changes in STS (Zhang et al., 2020; Crane and Paller, 2022) and SHOX (Hirschfeldova et al., 2012; Ogushi et al., 2019) genes were associated with multiple genetic defects, and chromosomal alterations involved in those genes were defined as pathogenic CNVs. Determining the parent-of-origins of the deletions/duplications is critical for the prenatal diagnosis of sex chromosomal abnormalities (Chen et al., 2020). In our study, we detected three cases of X or Y chromosomal deletions/duplications which were inherited from their parents with normal phenotypes and were defined as probably benign CNVs. However, because of the economic pressure and other concerns, most parents refused further testing. Also, 12 cases of sex chromosomal deletions/duplications defined as VOUS could not be further classified.

Overall, using SNP arrays, our results showed a detailed manifestation of sex chromosomal abnormalities in Fujian Province and validated some sex deletions/duplications using parent samples. Our analysis suggested that Xp22.2 duplications, Xp22.33 duplications, and Yp11.32 deletions were probably benign CNVs. However, some cases with mosaic sex chromosomal abnormalities should be further studied using other technologies. Moreover, parent-of-origins of the sex chromosomal abnormalities were critical for prenatal diagnosis and should be used more widely.

## Data Availability

The datasets presented in this study can be found in online repositories. The names of the repository/repositories and accession number(s) can be found at https://www.ncbi.nlm.nih.gov/, GSE208389.
